# Using total plasma triacylglycerol to assess hepatic *de novo* lipogenesis as an alternative to VLDL triacylglycerol

**DOI:** 10.1080/03009734.2020.1739789

**Published:** 2020-03-25

**Authors:** Leanne Hodson, Sion A. Parry, Thomas Cornfield, Catriona Charlton, Wee Suan Low, Charlotte J. Green, Fredrik Rosqvist

**Affiliations:** a Oxford Centre for Diabetes, Endocrinology and Metabolism, Churchill Hospital, University of Oxford, Oxford, UK;; b National Institute for Health Research Oxford Biomedical Research Centre, Oxford University Hospitals Foundation Trust, Oxford, UK;; c Department of Public Health and Caring Sciences, Clinical Nutrition and Metabolism, Uppsala University, Uppsala, Sweden

**Keywords:** *De novo* lipogenesis, DNL, hepatic, human, triacylglycerol, VLDL

## Abstract

**Background:** Hepatic *de novo* lipogenesis (DNL) is ideally measured in very low-density lipoprotein (VLDL)-triacylglycerol (TAG). In the fasting state, the majority of plasma TAG typically represents VLDL-TAG; however, the merits of measuring DNL in total plasma TAG have not been assessed. This study aimed to assess the performance of DNL measured in VLDL-TAG (DNL_VLDL-TAG_) compared to that measured in total plasma TAG (DNL_Plasma-TAG_).

**Methods:** Using deuterated water, newly synthesised palmitate was determined in fasting plasma VLDL-TAG and total TAG in 63 subjects taking part in multiple studies resulting in *n* = 123 assessments of DNL (%new palmitate of total palmitate). Subjects were split into tertiles to investigate if DNL_Plasma-TAG_ could correctly classify subjects having ‘high’ (top tertile) and ‘low’ (bottom tertile) DNL. Repeatability was assessed in a subgroup (*n* = 16) with repeat visits.

**Results:** DNL_VLDL-TAG_ was 6.8% (IQR 3.6–10.7%) and DNL_Plasma-TAG_ was 7.5% (IQR 4.0%−11.0%), and the correlation between the methods was *r_s_* = 0.62 (*p* < 0.0001). Bland–Altman plots demonstrated similar performance (mean difference 0.81%, *p* = 0.09); however, the agreement interval was wide (−9.6% to 11.2%). Compared to DNL_VLDL-TAG_, 54% of subjects with low DNL were correctly classified, whilst 66% of subjects with high DNL were correctly classified using DNL_Plasma-TAG_. Repeatability was acceptable (i.e. not different) at the group level, but the majority of subjects had an intra-individual variability over 25%.

**Conclusion:** DNL in total plasma TAG performed similarly to DNL in VLDL-TAG at the group level, but there was large variability at the individual level. We suggest that plasma TAG could be useful for comparing DNL between groups.

## Introduction

*De novo* lipogenesis (DNL), the process whereby non-lipid precursors (e.g. sugar/carbohydrate) are synthesised to fat, primarily occurs in the liver in humans (1). It is often suggested that enhanced DNL is related to increased risk of cardiometabolic diseases including type 2 diabetes, non-alcoholic fatty liver disease (NAFLD), and insulin resistance (2–6). As the major end product of DNL is the saturated fatty acid palmitate, hepatic DNL is commonly inferred from fatty acid composition in various circulating lipid fractions (e.g. phospholipids and erythrocytes) (1–5). We recently demonstrated, in a large group of healthy men and women, that fatty acid markers were poor proxies for isotopically assessed fasting hepatic DNL during habitual dietary conditions (7). The gold standard method for measuring hepatic DNL in humans involves stable-isotope tracers, where the appearance of the stable-isotope label (from deuterated water [^2^H_2_O] or [^13^C]acetate) is measured in palmitate in very low-density lipoprotein (VLDL) triacylglycerol (TAG; equivalent to the commonly used term ‘triglycerides’) (8). However, the process of isolating VLDL using ultracentrifugation is costly, time-consuming, has a low throughput, and can require up to 3 ml of plasma, depending on the ultracentrifugation method used. Thus, it is not feasible to assess hepatic DNL in larger-scale studies using the gold standard method. A method whereby VLDL isolation is not required could make the measurement of DNL more accessible. Total plasma TAG (instead of VLDL-TAG) is seldom used to assess DNL, but in the fasting state a large proportion (∼60–75%) of plasma TAG represents VLDL-TAG (9,10). Thus, fasting DNL assessed in total plasma TAG may be reflective of fasting DNL assessed in VLDL-TAG. Using total plasma TAG would offer several advantages (in terms of cost, throughput, plasma volume, and time), but a direct comparison between DNL assessed in total plasma TAG and VLDL-TAG has not previously been performed.

Here, we utilised stable-isotope methodology (deuterated water) to measure newly synthesised palmitate in VLDL-TAG (‘gold standard hepatic DNL’) and compared this to newly synthesised palmitate in total plasma TAG (‘poor man’s DNL’).

## Subjects and methods

### Subjects

Subjects were recruited from the Oxford Biobank (www.oxfordbiobank.org.uk) or were from the Oxfordshire area. All volunteers were non-diabetic and free from any known metabolic disease, were not taking medication known to affect lipid or glucose metabolism, and did not consume alcohol above recommended UK limits (≤14 units per week; 1 unit equals 8 g of pure alcohol). The data and plasma used for the present analysis were from subjects taking part in published or ongoing trials with different primary aims (ClinicalTrials.gov Identifiers: NCT03090347, NCT02478541, NCT01936779, and NCT03145350). Data and plasma from *n* = 63 individuals (46 males, 17 females) were used. Because most individuals (*n* = 60; 44 males, 16 females) took part in two separate study visits (pre–post intervention) and data and plasma from both study visits were used when available, a total of *n* = 123 data points were used for the current analysis. Prior to study days subjects were encouraged to refrain from strenuous exercise and alcohol intake and to consume a low-fat meal the evening before measurement in order to standardise the last meal. Subjects had fasted ≥10 h prior to collection of blood samples. A subgroup of *n* = 16 subjects who were not involved in any intervention were asked to maintain their habitual lifestyle for 4 weeks before repeated measurements were performed. All studies were approved by the respective Research Ethics Committees (reference numbers 12/SC/0267, 15/NS/0117, 17/NE/0031, and 16/NW/0751), and all subjects provided written informed consent.

### Measurements of DNL

Deuterated water (3 g/kg body water) was administered the evening prior to measurement. A fasting whole-blood sample was collected the following morning, and plasma was immediately separated by centrifugation at 4 °C. Separation of the VLDL-rich fraction (S_f_20–400) was made by sequential flotation using density gradient ultracentrifugation (11,12). Lipids in the VLDL-rich fraction and total plasma were extracted using chloroform-methanol, and TAG was separated by solid-phase extraction (12). Fatty acid methyl esters were prepared using methanolic sulphuric acid, and fatty acid relative abundance (mol%) was determined by gas chromatography (12). DNL was assessed based on the incorporation of deuterium in plasma water into VLDL-TAG palmitate and plasma TAG palmitate using GC-MS, monitoring ions with mass-to-charge ratios of 270 (M + 0) and 271 (M + 1) (13). Background isotopic enrichment in plasma water and VLDL-TAG was measured in a fasting blood sample taken before subjects consumed deuterated water.

### Clinical chemistry

Blood was collected into heparinised tubes, and plasma was separated by centrifugation at 4 °C. Plasma glucose (Werfen, Warrington, UK, 18250840; CV 1.3%), triacylglycerol (Werfen, 18255640; CV 1.7%), total cholesterol (Werfen, 18250540; CV 1.7%), and HDL cholesterol (Randox, CH2652; CV 2.5%) were analysed enzymatically (ILab 600/650 clinical chemistry; Werfen), and insulin was analysed by radioimmunoassay (Millipore, UK).

### Statistics

Data were analysed using JMP 13.1.0 (SAS Institute Inc.). The correlation between the two assessments was analysed using Spearman’s rank correlation. A Bland–Altman plot (difference plot) was used to analyse agreement between the two assessments and for analysing repeatability. Subjects were split into tertiles (based on DNL) in order to investigate if DNL assessed in total plasma TAG could correctly classify/distinguish subjects having ‘high’ (top tertile) and ‘low’ (bottom tertile) DNL. Using tertiles was an arbitrary choice, but has the benefits of creating distinct groups without compromising group sizes too much. An alluvial plot was used to visualise misclassification when using total plasma TAG.

## Results

Characteristics of the 63 individuals are given in Table 1.

**Table 1.** Subject characteristics.

Characteristics	Mean ± SD
Age, years	46 ± 6
Sex, M/F	46/17
BMI	29.3 ± 3.9
Glucose, mmol/L	5.5 ± 0.9
Insulin, mU/L	13.5 ± 8.5
Triglycerides, mmol/L	1.5 ± 0.9
Total cholesterol, mmol/L	5.1 ± 1.0
HDL cholesterol, mmol/L	1.2 ± 0.4

Data (mean ± SD) are based on baseline/first visit data (*n* = 63); follow-up/second visit data were similar (data not shown).

### Association and agreement between DNL_VLDL-TAG_ and DNL_Plasma-TAG_

**Figure 1.**
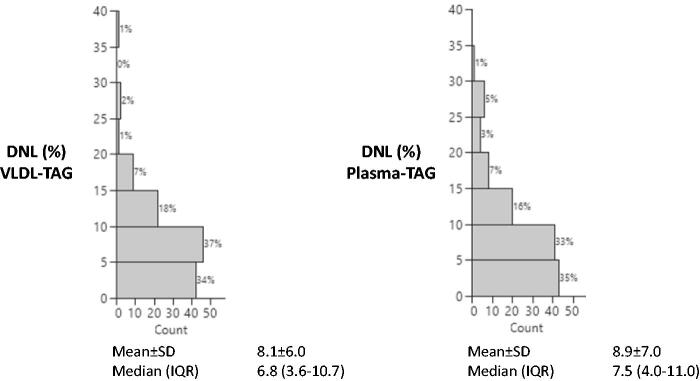
Histograms showing the distributions, means, and medians of DNL_VLDL-TAG_ and DNL_Plasma-TAG_. *n* = 123.

**Figure 2.**
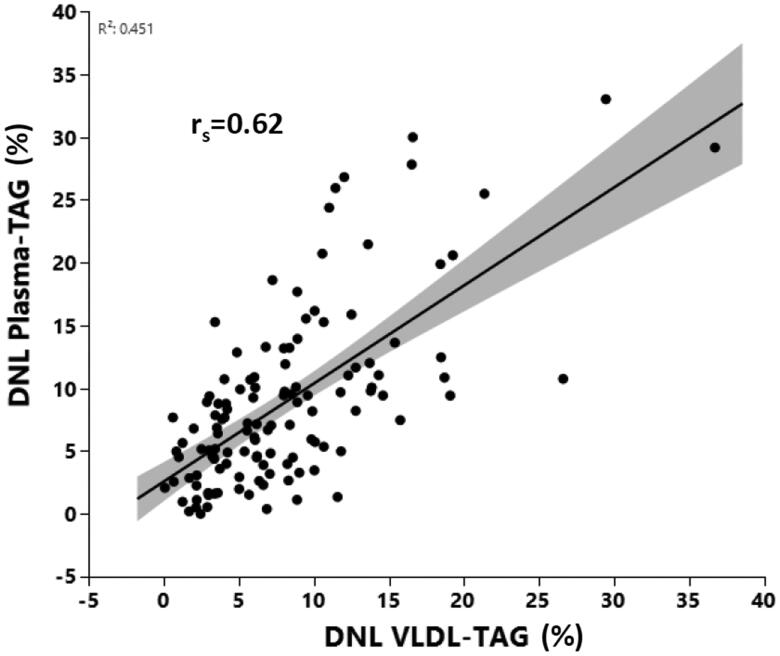
Correlation between DNL_VLDL-TAG_ and DNL_Plasma-TAG_. *n* = 123.

**Figure 3.**
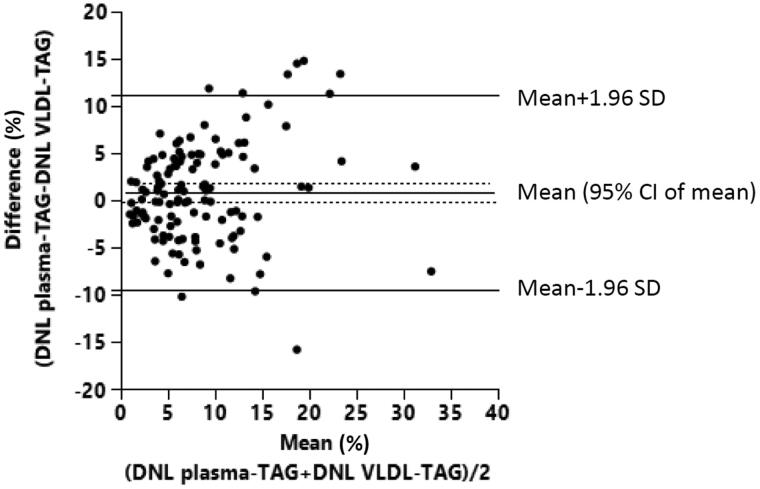
Bland–Altman plot showing the agreement between DNL_VLDL-TAG_ and DNL_Plasma-TAG_. *n* = 123.

The distribution of DNL_VLDL-TAG_ and DNL_Plasma-TAG_ was similar, with the medians being 6.8% (IQR 3.6 − 10.7%) and 7.5% (IQR 4.0−11.0%), respectively (Figure 1). There was a strong positive correlation between the two methods (Figure 2). Using a Bland–Altman plot to compare the two methods, it was found that although the two methods performed similarly (mean difference 0.81%, *P* = 0.09), the agreement interval was wide (−9.6 to 11.2%) (Figure 3).

**Figure 4.**
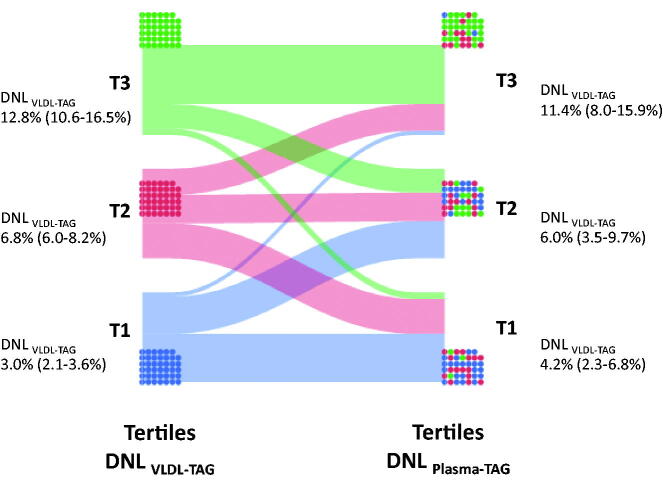
Alluvial plot with subjects split into tertiles based on DNL_VLDL-TAG_ and DNL_Plasma-TAG_, showing misclassification of subjects when using DNL_Plasma-TAG_. Median (IQR) DNL_VLDL-TAG_ is shown beside respective tertile. Coloured dots represent composition of the tertile; e.g. red dots (subjects) in the top tertile of DNL_Plasma-TAG_ actually belong to the middle tertile when using DNL_VLDL-TAG_.

We classified subjects into tertiles based on DNL_VLDL-TAG_ and DNL_Plasma-TAG_ and found the medians for the respective tertiles to be 3.0% (2.1−3.6%), 6.8% (6.0−8.2%), and 12.8% (10.6−16.5%) for DNL_VLDL-TAG_ and 4.2% (2.3−6.8%), 6.0% (3.5−9.7%), and 11.4% (8.0−15.9%) for DNL_Plasma-TAG_ (Figure 4). Although there was a strong positive correlation between methods, 50% of individuals were misclassified from their DNL_VLDL-TAG_ tertile when classified using DNL_Plasma-TAG_. For the top tertile of DNL_VLDL-TAG_ 66% (27/41) were correctly classified, whereas this number was 32% (13/41) for the middle tertile and 54% (22/41) for the bottom tertile (coloured points in Figure 4).

### Repeatability of DNL_VLDL-TAG_ and DNL_Plasma-TAG_

**Figure 5.**
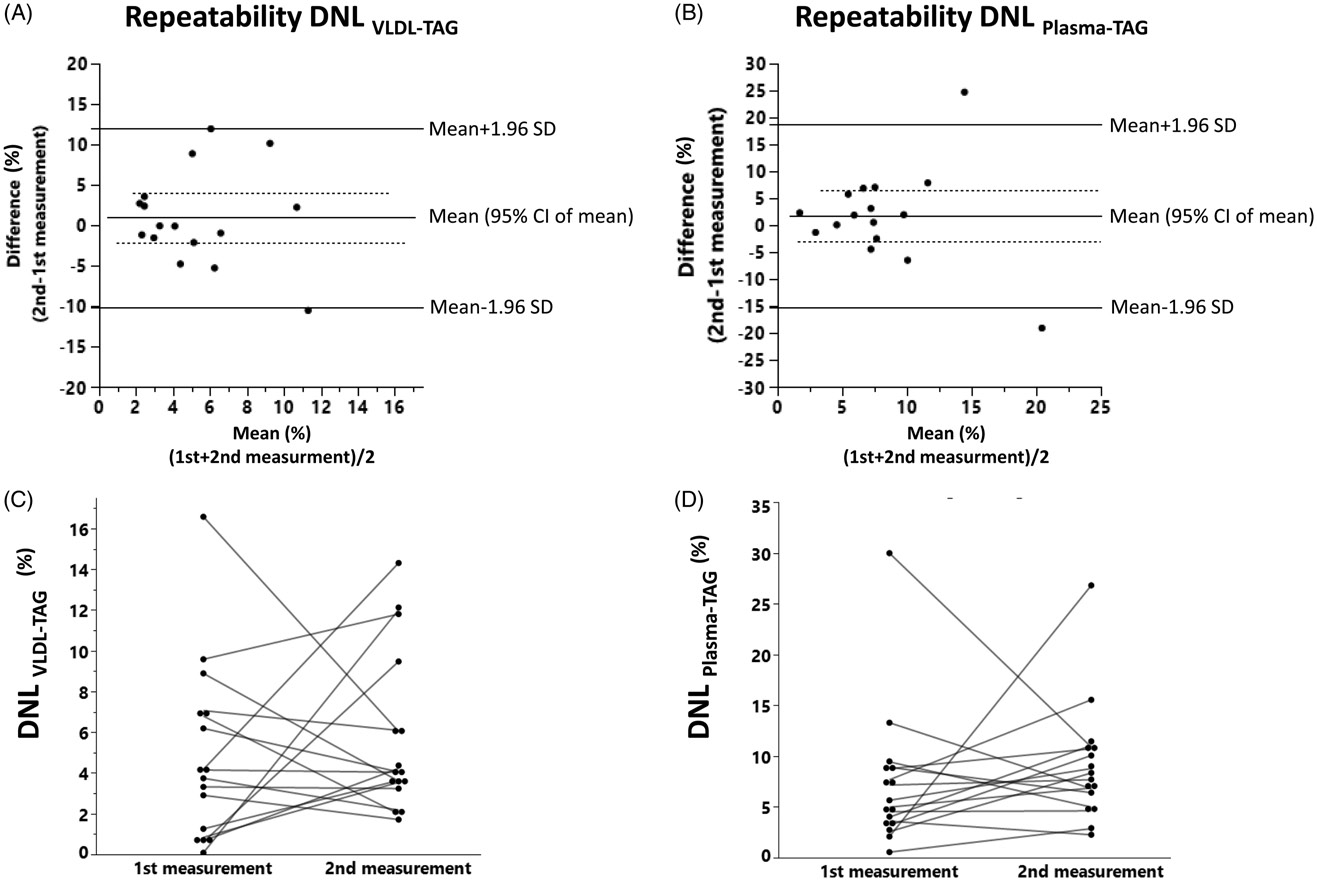
Repeatability of DNL measured in VLDL-TAG and Plasma-TAG. (A) Bland-Altman plot showing mean and difference of the two measurements of DNL in VLDL-TAG; (B) Bland-Altman plot showing mean and difference of the two measurements of DNL in Plasma-TAG; (C) Matched-pair plot showing the individual differences between first and second measurement of DNL in VLDL-TAG; (D) Matched-pair plot showing the individual differences between first and second measurement of DNL in Plasma-TAG. *n* = 16.

Repeatability of DNL_VLDL-TAG_ and DNL_Plasma-TAG_ was assessed in 16 subjects (8 males, 8 females) not taking part in any intervention but with repeated measurements separated by 4 weeks. For DNL_VLDL-TAG_, the first and second measurements were similar (*p* = 0.53) at the group level with a mean difference of 0.94%; however, 12/16 subjects had an intra-individual difference of more than 25% (Figure 5(A,B)). For DNL_Plasma-TAG_, the first and second measurements were similar (*p* = 0.44) at the group level with a mean difference of 1.8%; however, 13/16 subjects had an intra-individual difference of more than 25% (Figure 5(C,D)).

## Discussion

We assessed the use of plasma TAG to measure hepatic DNL and compared this to VLDL-TAG in a large sample of metabolically healthy adult humans. We found that plasma TAG performed similarly in terms of mean and distribution compared to VLDL-TAG. Although misclassification was evident at the individual level, plasma TAG worked well for determining DNL at the group level and performed best in individuals having higher DNL. Using a less intensive method of measuring hepatic DNL (based on plasma and not requiring ultracentrifugation for isolation of VLDL) could aid our understanding of the role hepatic DNL has in health and disease.

We have recently demonstrated that fatty acid composition of circulating TAG was not a good reflection of isotopically measured DNL in healthy humans (7). Therefore, in the present work, we used deuterated water to assess DNL in circulating total plasma TAG and compared this with DNL in isolated VLDL-TAG. As the majority (∼60–75%) of plasma TAG in the fasting state typically is from VLDL-TAG (9,10), it would be reasonable to assume that DNL in plasma TAG may be reflective of DNL in VLDL-TAG. Using plasma TAG instead of VLDL-TAG potentially increases the accessibility for assessing hepatic DNL as well as making it cheaper, as VLDL-TAG isolation would not be required and plasma could be retrospectively analysed in batches instead of continuously during an ongoing study. Compared to simple fatty acid markers of hepatic DNL (as reported in our previous paper [7]), the assessment reported here (DNL_Plasma-TAG_) showed 2–3-fold stronger associations with DNL_VLDL-TAG_. Furthermore, the DNL_Plasma-TAG_ and DNL_VLDL-TAG_ assessments were shown to perform similarly in terms of mean (differing only 0.81%) and distribution of DNL. However, the agreement interval between the two assessments was wide (∼21 percentage points), and overall 50% of subjects were misclassified using plasma TAG, suggesting that plasma TAG is not an optimal substitute and likely to be more useful for separating individuals with high and low DNL, rather than investigating subtle individual changes, such as those that may be observed in response to an intervention. When analysing repeatability in a subgroup, both DNL_VLDL-TAG_ and DNL_Plasma-TAG_ were found to be repeatable at the group level; however, the majority of subjects had a large or very large intra-individual variability. Variability of DNL at the individual level has not previously been reported and may be due to both methodological and biological reasons, for example: (i) subjects did not receive a standardised diet the days preceding measurement, hence both macronutrient composition and energy balance were uncontrolled for; (ii) deuterium labelling may have been too low (increasing the noise) or too short (susceptible to intrahepatic fatty acid turnover) (6); and (iii) factors regulating intrahepatic fatty acid partitioning may have changed between measurements, potentially affecting the amount of secreted VLDL-TAG. It would be valuable to investigate intra-individual variability during more tightly controlled standardisation settings in order to disentangle methodological and biological reasons.

In line with the above, another potential reason for the discrepancy between DNL_VLDL-TAG_ and DNL_Plasma-TAG_ is that the proportion of VLDL-TAG in total TAG may differ between individuals; i.e. DNL_Plasma-TAG_ should be more reflective of DNL_VLDL-TAG_ in subjects where a larger proportion of plasma TAG is VLDL-TAG. The proportion of VLDL-TAG in plasma TAG would be an unknown factor in most cases and thus constitutes a potential limitation of using plasma TAG.

Limitations of the current study include the relatively low number of subjects used for repeatability analyses as well as not controlling for background diet in this analysis. Furthermore, we compared the assessments in the fasting state only, and further studies are needed to investigate if plasma TAG could be used in the postprandial state. It could be speculated, however, that plasma TAG may potentially underestimate DNL postprandially due to the presence of chylomicrons and chylomicron remnants in circulation. A strength of the study is the large sample size with isotopically measured hepatic DNL. Furthermore, subjects were metabolically healthy and included both males and females. However, further work would need to be performed in patient populations (e.g. NAFLD) and across a wider phenotype.

In conclusion, DNL measured in total plasma TAG is more reliable than simple fatty acid markers and performed similarly to DNL measured in VLDL-TAG at the group level, but demonstrated large inconsistencies at the individual level. This simplified method could be useful for distinguishing between groups having higher and lower DNL (e.g. based on tertiles) but not used as a continuous variable.
